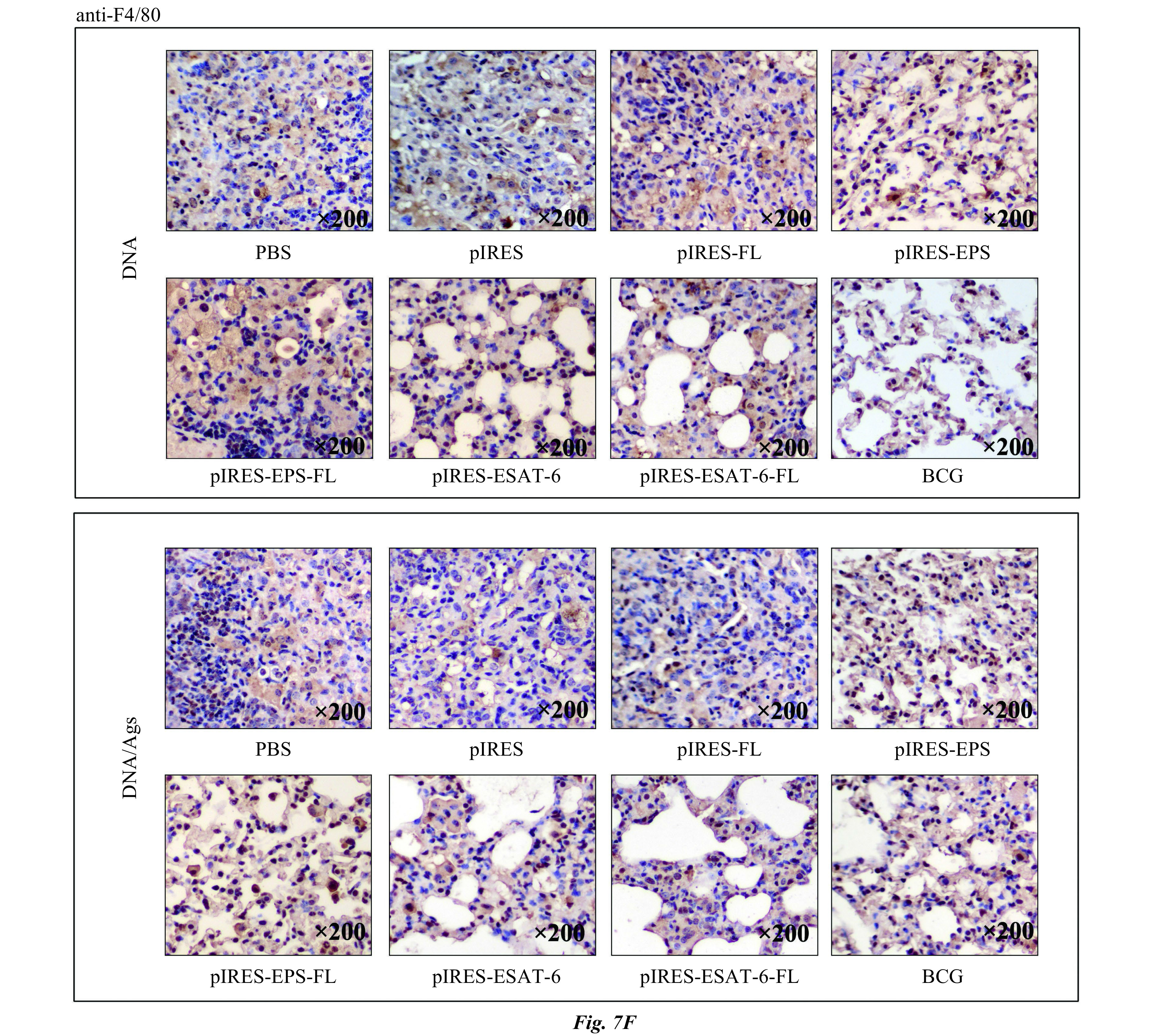# Author Correction: A novel recombinant DNA vaccine encoding *Mycobacterium tuberculosis* ESAT-6 and FL protects against *Mycobacterium tuberculosis* challenge in mice

**Published:** 2022-11

**Authors:** Qingtao Jiang, Jing Zhang, Xia Chen, Mei Xia, Yanlai Lu, Wen Qiu, Ganzhu Feng, Dan Zhao, Yan Li, Fengxia He, Guangyong Peng, Yingwei Wang

**Affiliations:** 1 Department of Microbiology and Immunology, Nanjing Medical University, Nanjing, Jiangsu 211166, China; 2 Division of Infectious Diseases, Allergy and Immunology, Department of Internal Medicine, Saint Louis University School of Medicine, St. Louis, Missouri 63104, USA

Correction to: *Journal of Biomedical Research* https://doi.org/10.7555/JBR.27.20120114,published on 30 September 2013.

We apologize for the misused images in ***Fig. 7*** in our article published online on 10 May 2012. The ***Fig. 7*** by HE staining and immunohistochemistry were partially misused because of our carelessness. Because we couldn't find the original pictures acquired 11 years ago, thus HE and IHC experiments were repeated by our previously reserved wax blocks. Now, the pictures taken by microscope imaging system in each group were collected and combined, respectively. The correct version of ***Fig. 7C***, ***E***, and ***F*** are shown below. This correction does not affect the conclusion reported in the study.

**Figure7:**
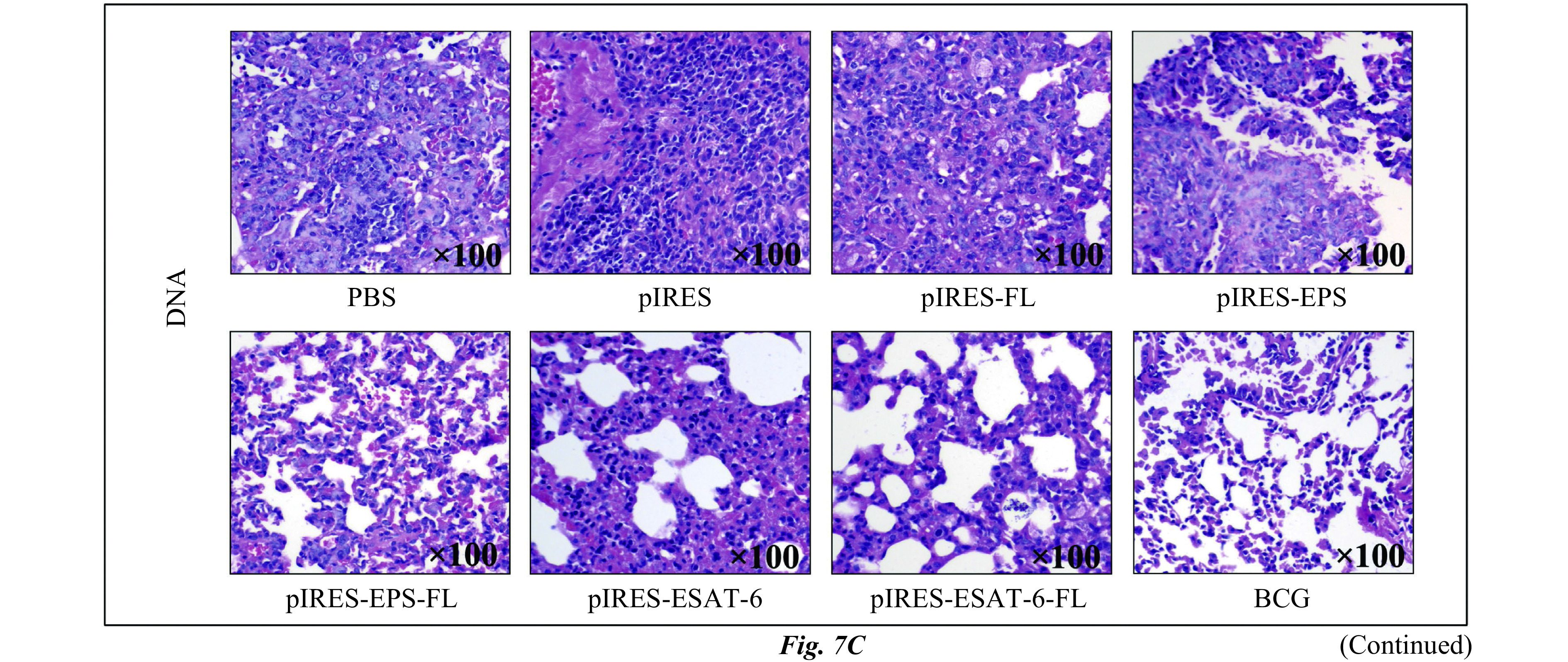


**Figure7-1:**
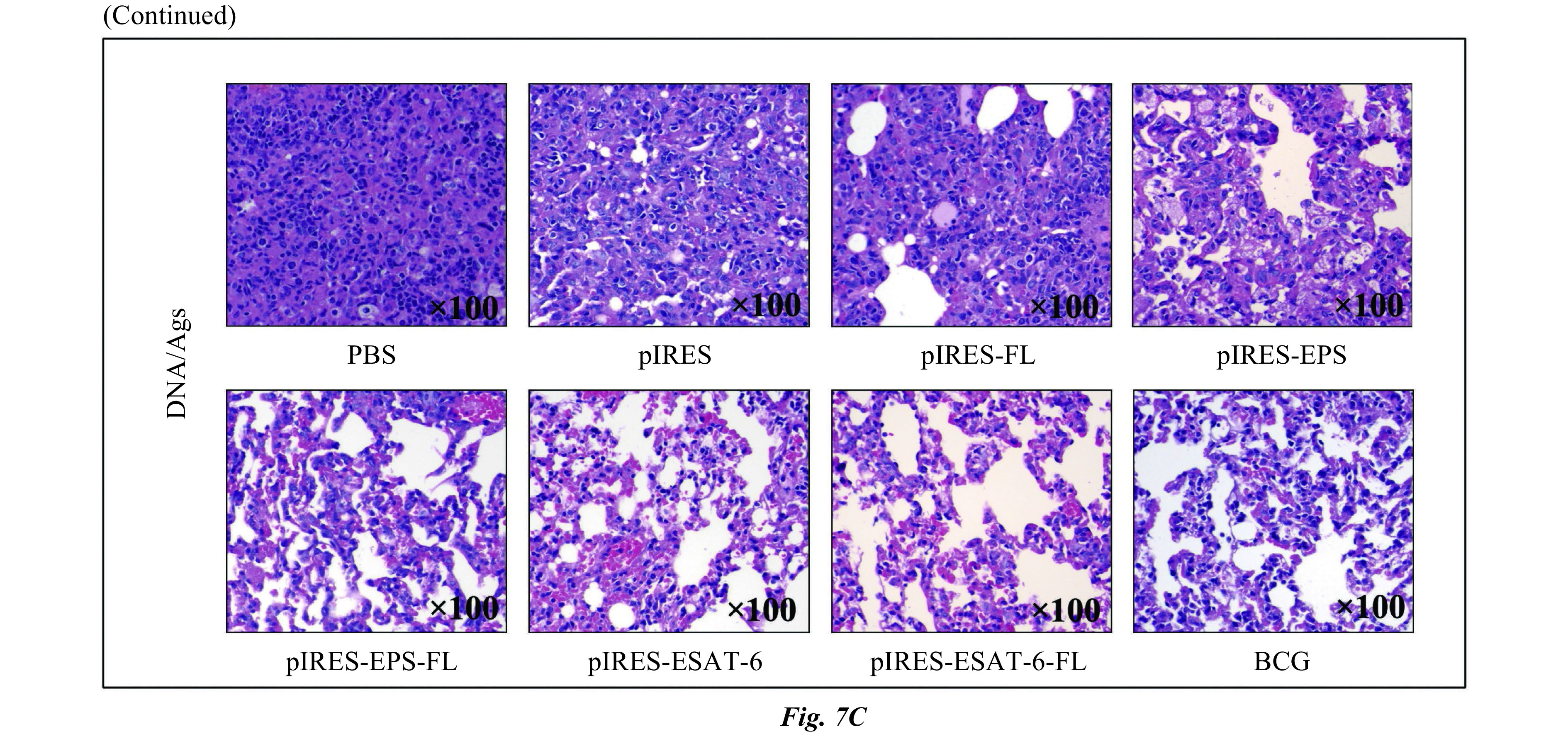


**Figure7-2:**
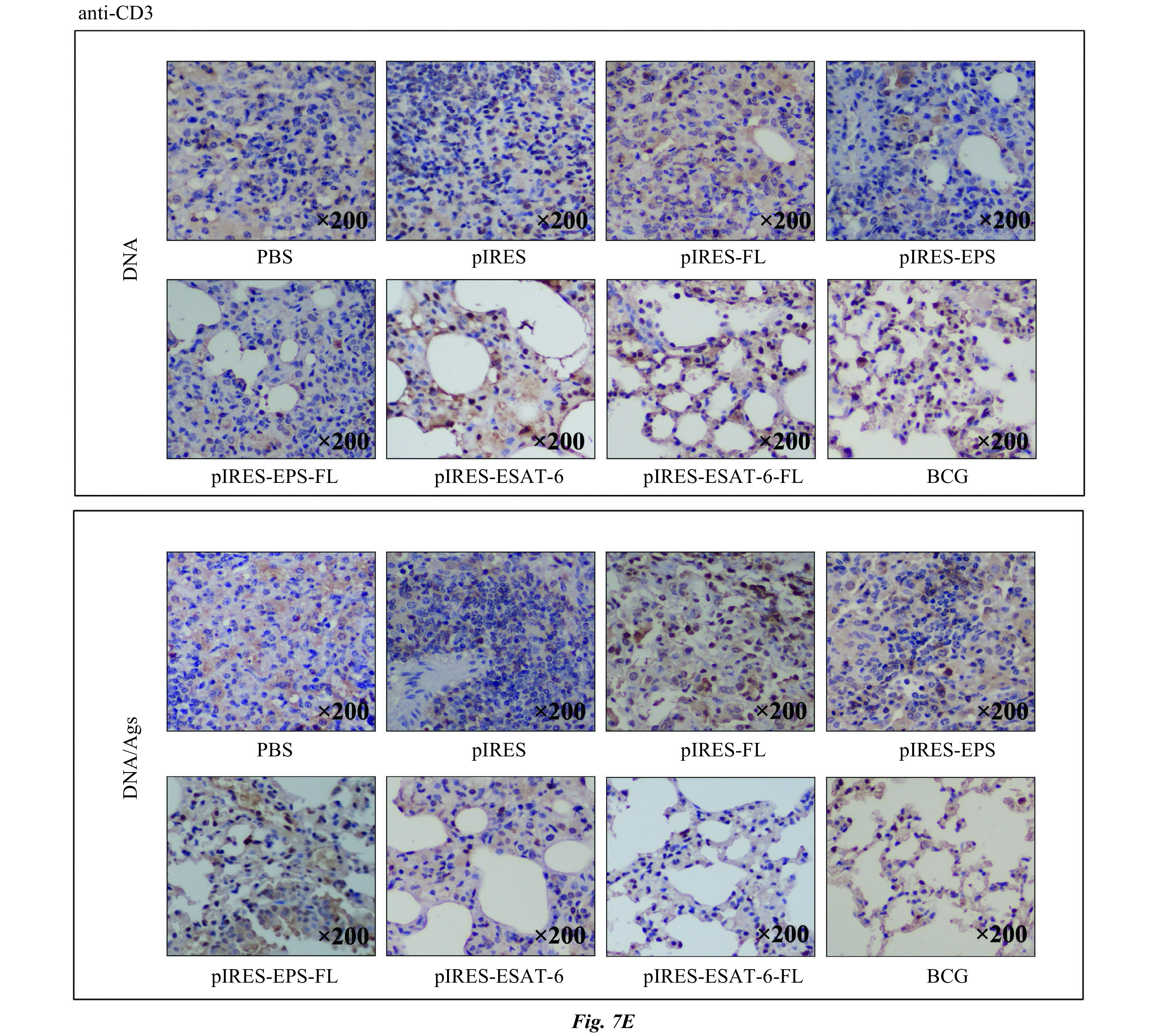


**Figure7-3:**